# The Roots of Plant Frost Hardiness and Tolerance

**DOI:** 10.1093/pcp/pcz196

**Published:** 2019-10-18

**Authors:** Valentin Ambroise, Sylvain Legay, Gea Guerriero, Jean-Francois Hausman, Ann Cuypers, Kjell Sergeant

**Affiliations:** 1 Environmental Research and Innovation (ERIN) Department, Luxembourg Institute of Science and Technology (LIST), 5 Avenue des Hauts-Fourneaux, L-4362 Esch/Alzette, Luxembourg; 2 Centre for Environmental Sciences, Hasselt University, Agoralaan Building D, B-3590 Diepenbeek, Belgium

**Keywords:** Abiotic stress, Cold acclimation, Frost, Hardiness, Roots, Tolerance

## Abstract

Frost stress severely affects agriculture and agroforestry worldwide. Although many studies about frost hardening and resistance have been published, most of them focused on the aboveground organs and only a minority specifically targets the roots. However, roots and aboveground tissues have different physiologies and stress response mechanisms. Climate models predict an increase in the magnitude and frequency of late-frost events, which, together with an observed loss of soil insulation, will greatly decrease plant primary production due to damage at the root level. Molecular and metabolic responses inducing root cold hardiness are complex. They involve a variety of processes related to modifications in cell wall composition, maintenance of the cellular homeostasis and the synthesis of primary and secondary metabolites. After a summary of the current climatic models, this review details the specificity of freezing stress at the root level and explores the strategies roots developed to cope with freezing stress. We then describe the level to which roots can be frost hardy, depending on their age, size category and species. After that, we compare the environmental signals inducing cold acclimation and frost hardening in the roots and aboveground organs. Subsequently, we discuss how roots sense cold at a cellular level and briefly describe the following signal transduction pathway, which leads to molecular and metabolic responses associated with frost hardening. Finally, the current options available to increase root frost tolerance are explored and promising lines of future research are discussed.

## Introduction

Frost is a major meteorological factor impacting agriculture and the agroforestry economy in temperate climates (Snyder and de Melo-Abreu 2005, Papagiannaki et�al. 2014), as well as in many subtropical regions (Snyder and de Melo-Abreu 2005), determining the distribution range of many plants and crops. Freezing stress threatens plants integrity by inducing the growth of ice crystals within the plant tissues. However, many plant species from temperate and cold climates can increase their ability to withstand freezing temperatures after being exposed to environmental stimuli such as low temperatures and short days. This complex process, called cold acclimation or frost hardening, is associated with biochemical and physiological changes that ultimately lead to changes in gene expression, osmolyte accumulation and lipid bilayer composition.

Whereas it is widely documented that the climate is changing, frost damage will likely not disappear in a globally warmer climate and may even become more problematic in some regions of the world (Gu et�al. 2008). Indeed, while global warming causes milder winter temperatures on average, it also destabilizes polar vortices, thereby increasing temperature variance and the probability of extreme weather events (Francis and Skific 2015). Furthermore, plants harden less under warmer temperatures, making them less resistant to frost in winter, even if milder (Murray et�al. 1994). Taken together, these factors will greatly affect plant distribution and productivity. In most regions, milder winter temperatures will expand the distribution range of species and also increase the probability of early- and late-frost events (Schwartz et�al. 2006). During the last 60 years, global warming hastened budbreak and frost dehardening/deacclimation by >1 d per decade (Schwartz et�al. 2006, Man et�al. 2009). However, plants are the most frost sensitive just after dehardening and during budbreak, as they mobilize reserve resources accumulated during cold acclimation to resume growth (Kalberer et�al. 2006). This conjunction of advanced budbreak and late frost caused serious damage in Eastern US forests during the springs of 2007 (Gu et�al. 2008, Augspurger 2009) and 2010 (Hufkens et�al. 2012). Similarly, in some locations of Southern Europe, climate change and the increased frequency of late-frost events shortened the frost-free season by >50 d since 1975 (Lavalle et�al. 2009). In addition, to avoid the deleterious effects of summer drought on grain filling, farmers have to sow crops earlier, increasing the need of frost-resistant cultivars and winter varieties (Rezaei et�al. 2015). Furthermore, it is expected that global warming will shift species distribution to higher latitudes, sometimes by >250 km, where they will be more exposed to extreme weather events (Park et�al. 2016).

The precise impact of the increase in CO_2_ level accompanying the rise of temperatures is currently a matter of debate. Studies indicate that higher CO_2_ concentrations influence the frost resistance in a species-dependent manner. In addition to these changes in maximum frost hardiness, increased CO_2_ concentrations have been shown to slow down hardening (Barker et�al. 2005) and hasten dehardening (Murray et�al. 1994, Repo et�al. 1996) of some species, making them more vulnerable to cold spells.

Most studies on frost resistance mechanisms focus on the aboveground organs (Lee et�al. 2009, Sm�kalov� et�al. 2014). This originates from the difficulty to study roots in their natural environment and the fact that frost damage to the roots appears as a minor concern, as roots are less exposed due to the insulation provided by the soil (Weiser 1970, Colombo et�al. 1995, Regier et�al. 2010). However, aboveground and belowground organs have distinct response mechanisms, even at a molecular level (Bigras and Dumais 2005, Hashimoto and Komatsu 2007, Ryypp� et�al. 2008). As an example, in *Arabidopsis thaliana*, 86% of the cold-induced transcriptome changes are not shared between roots and aboveground organs (Kreps et�al. 2002).

Belowground, frost mainly damages and kills fine roots, which are crucial for water and mineral absorption. This costs both time and nonstructural carbohydrate reserves to regrow them once conditions improve. This is at the expense of aboveground productivity (Gaul et�al. 2008, Man et�al. 2009), generally the valued plant parts. Frost also reduces agroforestry productivity by damaging container-grown seedlings, as their roots are exposed to lower temperatures in containers than they would be in nature (Bigras and Dumais 2005). Climate change may also increase root frost damage through a reduction of the snow cover and therefore of soil insulation (Comerford et�al. 2013) (Fig.�1). Gaul et�al. (2008) recorded that the temperature of the soil’s top 20 cm, where approximately 70% of the fine roots are found, could decrease by up to 5.5�C (from 0.2�C to −5.3�C) after manually removing the snow cover in a spruce forest growing on an haplic podzol in South Eastern Germany. Similarly, Cleavitt et�al. (2008) reported that in the absence of snow cover, frost propagated 40 cm deeper in a hardwood forest in North Eastern USA (New Hampshire). In addition, roots lacking snow insulation are more exposed to freeze–thaw cycles. All these can result in significant damage to fine roots, leading to a decrease in the fine root biomass by 50% in boreal forests (Kreyling et�al. 2012) and by up to 80% in container-grown *Pinus sylvestris* seedlings (Sutinen et�al. 1996).

**Fig. 1:**
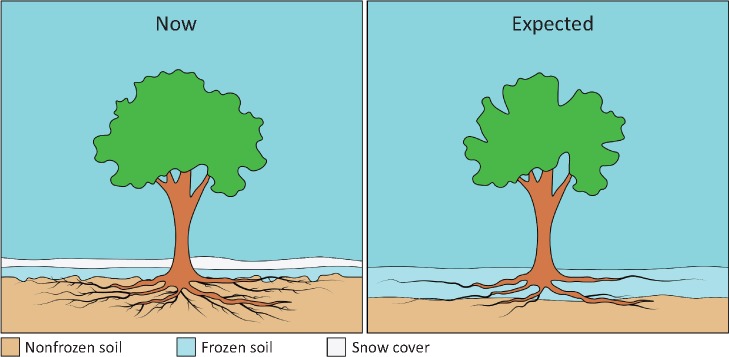
Soil freezing patterns expected in the future. In the future, increased temperature could lead to a decreased snow cover, leading to a less insulated soil and lower soil temperatures. This would decrease the fine root biomass by up to 50% in summer, highly reducing the new aboveground biomass.

The purpose of this review is to present the current knowledge concerning the impact of frost conditions on roots. The freezing mechanism and the origins of frost damage in plant roots are discussed. Then, we present the environmental signals inducing cold tolerance/frost hardiness of the roots, as well as signal transduction pathway and physiological changes taking place during acclimation. Finally, we discuss some leads to improve root frost hardiness.

## Freezing Stress and Sources of Frost Damage

Plants and plant tissues developed the following two contrasting strategies to cope with freezing stress: tolerate it and avoid it (Burke et�al. 1976, Levitt 1980). Freezing tolerance allows plants and/or their tissues to tolerate the presence of ice in extracellular spaces and the ensuing dehydration stress. On the other hand, freeze avoidance relies on the ability plants to avert the formation of interstitial ice crystals. Although these two strategies are clearly different, they can be found simultaneously in the same plant.

Frost-avoiding tissues can avoid freezing either by insulating themselves from the cold temperatures or by limiting the formation of ice crystals in their tissues by keeping water liquid below 0�C. The ability to keep water under a liquid state at freezing temperatures is called supercooling (or undercooling) and relies on the fact that the freezing point of pure water, also known as the ice homogeneous nucleation temperature, is at approximately −40�C (Rasmussen and MacKenzie 1972). However, in nature, water usually freezes at temperatures just below 0�C. This is because in nature, water is nearly always found in association with salts or colloids, under the form of a solution. Some of these salts and colloids have the capacity to act as ice seeds and induce ice nucleation. These particles are called ice-nucleating agents (INAs) and induce heterogeneous nucleation. Within the root environment, some INAs are synthesized by frost-tolerant plants in their extracellular space to allow the initiation of ice nucleation in the apoplast rather than in the symplast. In addition to supercooling, frost-avoiding tissues can further decrease the ice nucleation temperature by synthesizing osmolytes in their fluids, which reduces the water freezing point by 1.86�C per mole of solute (Zachariassen and Kristiansen 2000). Because of the large amount of solute required to significantly reduce the freezing point, the actual freezing point depression that can be achieved in planta is of only 1–2�C (Levitt 1980). Antifreeze proteins (AFPs), i.e. proteins reducing the freezing point in a noncolligative way, can further reduce the freezing point by up to 0.4�C allowing plants to keep their internal fluids liquid under lower temperatures (Wisniewski and Fuller 1999).

Due to practical limitations associated with the study of roots in their natural environment, it is currently not known whether roots are frost tolerant or avoidant in their natural environment. However, based on ex situ experiments, it seems that they can be either one, depending on the species studied. The precise ice propagation path in frost-tolerant roots is unknown (Korhonen et�al. 2018).

Experiments on the supercooling capacity of aerial organs of monocot plants indicate that their ability to avoid ice formation is poor and it seems to be the same in their roots (Stier et�al. 2003, Livingston et�al. 2016). For example, in infrared thermography experiments on uprooted herbaceous monocots, ice nucleation was found to always occur in the roots first, followed by the crown and the leaves (Pearce and Fuller 2001, Stier et�al. 2003). Similar observation was made on in situ wheat (*Triticum aestivum*) by Livingston et�al. (2016). This is expected as soil contains a significant amount of INAs. These INAs can come from plants, fungi, bacteria, decaying matter and various dusts and salts (Murray et�al. 2012, Hill et�al. 2016). For instance, roots of *Artemisia tridentata* have been found to produce INAs that are able to initiate ice nucleation at a temperature between −5�C and −12�C (Hill et�al. 2016). Some soil fungi produce INAs that are able to initiate ice nucleation at similar temperatures (Morris et�al. 2013, Hill et�al. 2016), and some soil pathogenic bacteria, such as *Pseudomonas syringae*, produce INAs that nucleate water freezing at temperatures close to −2�C (Pearce 1999, Wisniewski et�al. 1999). In addition to these INAs, soils contain humic and fulvic acids, as well as sterols and membranes debris coming from decaying root material, which can nucleate ice formation at temperatures as high as −4�C (Hill et�al. 2016). Interestingly, Hill et�al. (2016) observed that soil contains approximately 10,000 times more INAs that are able to initiate ice nucleation at temperatures below −5�C than INAs that are able to initiate ice nucleation at temperatures above −5�C.

Similar to monocot roots, conifer roots seem to be frost tolerant. Indeed, Coleman et�al. (1992) observed that the ice nucleation temperature was several degrees higher than the LT_50_ in the roots of four conifer species, suggesting that they were able to withstand intercellular ice formation. Interestingly, it seems that mycorrhiza does not influence root frost hardiness (Korhonen et�al. 2015, Korhonen et�al. 2018).

In a study comparing the frost resistance mechanism of 14 Andean dicots found at high altitude, Squeo et�al. (1991) observed that their roots could either tolerate or avoid freezing damage. Although the authors note that soil-provided insulation is likely to ensure the survival of most roots, they mention that a frost-avoiding strategy (based on the insulation of supercooling) in such environment could be risky as supercooling and insulation of tissues can only be endured for a few hours.

On the contrary, roots of the *Olea europaea* could rely on a supercooling-based frost avoidance strategy. Using differential thermal analysis, Fiorino and Mancuso (2000) observed that the first and second exotherms (which correspond to the freezing of extracellular and intracellular fluids, respectively) of cold-acclimated coarse roots of *O. europaea* were of −7.4�C and −8.1�C. Similar results were observed in the fine roots. This indicates that although the roots of *O. europaea* seem to have a high frost-avoiding capacity, they die nearly as soon as ice nucleates within their tissues. However, the precise degree to which *O. europaea* roots are able to supercool their fluid under natural conditions is unknown as all these measures were carried out using excised and washed roots and, therefore, soil INAs were not present.

In addition to these two frost defense strategies, the roots of some perennial monocots have been found to senesce in a fashion similar to how deciduous plants shed their leaves in autumn. In a study on 26 species of perennial monocots, it was found that, depending on the species, the rate of winter survival of their roots was either over 85% or null (Nieman et�al. 2018). Interestingly, in plants with a null root survival rate, the roots did not die during the cold season but rather senesced during the cold acclimation period.

Although soil contains water, which can freeze and cause the formation of ice lenses and frost heaves, it seems that the main source of damage to the roots is direct cellular damage rather than mechanical damage (Cleavitt et�al. 2008). The main source of cellular damage during freezing stress depends on the strategy adopted to cope with frost. Frost-avoiding tissues are mainly damaged by intracellular ice formation, which leads to cell death. In supercooling-based frost-avoiding plants, as supercooled fluids eventually freeze, the freezing front propagates at a rate of several centimeters per second; the more the liquid is supercooled, the faster the propagation (Pearce and Fuller 2001). A high propagation speed can lead to the freezing of intracellular fluids and cell death. In uprooted *Lolium perenne* and *Poa supine*, Stier et�al. (2003) observed that the freezing front propagation was faster in roots than in leaves. However, the presence of INAs in soil and the insulation provided by soil (due to its high specific heat and the release of latent heat during the freezing of soil water) makes that studies on isolated roots may result in artifacts and that, in reality, ice nucleation in roots is initiated at temperatures close to 0�C. Therefore, ice would propagate a much slower speed than those observed with infrared thermal analysis on isolated roots. Nevertheless, this also indicates that while aboveground organs of monocots contain ice propagation barriers, it is not the case for their roots. Miller and Neuner (unpublished, in Wisniewski et�al. 2014) observed that the rhizoderm of seedlings does not have an ice propagation barrier. This could be different in mature dicot roots, which have endodermis and exodermis with fully functional Casparian strips and suberized cell walls. Hydrophobic layers are known to act as ice propagation barriers in aboveground organs (Kuprian et�al. 2016).

In contrast, in frost-tolerant roots, initial freezing occurs in the extracellular space (which contain INAs and lower solutes levels) at temperatures between −1.5�C and −3�C, depending on the cooling speed (Pearce and Fuller 2001, Stier et�al. 2003). When ice nucleates in the extracellular space, its water potential decreases, thereby provoking a net movement of water to the extracellular space. The plasmalemma is the main site of damage of the ensuing intense dehydration mainly through lamellar to hexagonal II phase transition (Strimbeck et�al. 2015) and expansion-induced lysis during freeze–thaw cycles (Xin and Browse 2000). Dehydration also promotes protein denaturation, cytorrhysis and xylem embolism (Charra-Vaskou et�al. 2016). It is noteworthy that roots are usually more exposed to frost-induced dehydration damage than aboveground organs. As the soil acts as a thermo-buffer, water in the roots stays liquid for a longer time than in the shoot and can then migrates to frozen extracellular space aboveground, which have a lower osmotic water potential (Bigras and Colombo 2001, Sakai and Larcher 1987).

All frost-exposed roots (and organs in general) also face mechanical disruption of their membranes due to the growth of extracellular icicles, as well as organ rupture provoked by ice expanding in their tissues (Burke et�al. 1976). In addition to these frost-specific injuries, chilling (exposure to nonfreezing temperatures) affects plants in various ways. It promotes membrane phase transition (from liquid ordered to gel-like solid) (Xin and Browse 2000, Pearce 2001), increases reactive oxygen species (ROS) production (Aroca et�al. 2005, Ruelland et�al. 2009), decreases the catalytic activity of enzymatic antioxidants and induces the formation of abnormal secondary mRNA structures (Sasaki et�al. 2007) (Fig.�2).

**Fig. 2:**
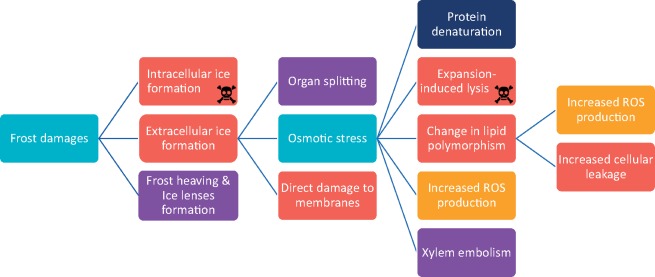
Sources of damage caused by low temperatures in plants. The main sources of damage during freezing temperatures are intracellular ice formation, which leads to cell death, and extracellular ice formation, which provokes osmotic stress. This osmotic stress mainly causes changes in lipid polymorphism, expansion-induced lysis, which leads to cell death, and an increased ROS production. Light blue, stressors; red, damage done to the membranes; yellow, imbalance in ROS homeostasis; dark blue, alterations in cellular metabolites and macromolecules; purple, physical damages. Skulls mean direct death of the cell via heavy damage to the membranes.

## Frost Hardiness

Frost hardiness is organ, species, variety and age dependent. In woody species, aboveground organs are hardier than roots and there is a frost hardiness gradient from buds (most hardy) to root tips (least hardy), with only a small decrease in frost hardiness at the air–soil interface (Zhao et�al. 1995, Ryypp� et�al. 2008, R�is�nen et�al. 2009). Fine roots are less hardy than lignified roots, e.g. *P. sylvestris* roots have an LT_50_ of −4.5�C and −9.1�C for fine and mature roots, respectively (Ryypp� et�al. 2008). Surprisingly, Korhonen et�al. (2015) observed that the nonacclimated needles of *P. sylvestris* exposed to various mycorrhizal and fertilization treatments were less frost hardy than the nonacclimated roots (−10.5�C and −8.5�C on average, respectively). In addition, while cold acclimation increased the frost hardiness of needles up to −14.2�C, the frost hardiness of roots decreased to −9.0�C with cold acclimation. This highlights the difference in response between roots and leaves. Therefore, there is a need to develop new methodologies and techniques specifically designed for studies focused on the root level (Korhonen et�al. 2018). Adult plants are hardier than juveniles and seedlings. However, the increase in frost hardiness is far less marked in the roots than in the shoots. The maximum frost hardiness of shoot cambium in *Quercus ilex* goes from −16�C during its first year to −25�C after 3 years, while for roots, the maximum frost hardiness goes from −6.5�C to −8�C during the same period (Sakai and Larcher 1987).

In herbaceous species belonging to the Poales order, aboveground organs are also hardier than belowground organs. The hardiest organs are renewal buds and leaf primordia, followed by older leaves and crown and finally roots that are the least resistant to freezing stress (Noshiro and Sakai 1979, Sakai and Larcher 1987). Hardened young *Poa pratensis* leaves are resistant up to −15�C, leaf primordia is resistant up to −17�C, winter buds are resistant up to −15�C, rhizome is resistant up to −13�C and fine roots are resistant up to −7�C (Noshiro and Sakai 1979). Although herbaceous plants can recover from important frost damage to their leaves and roots, damage to their crown (where meristems are found) results in reduced survival rate (Livingston et�al. 2016). In herbaceous dicots, roots could play a more important role. For instance, in pot-grown *Vicia faba* exposed to freezing temperatures, ultimate survival and shoot production are more closely related to roots’ survival than to shoots’ survival (Sallam et�al. 2015).

Several hypotheses have been proposed to explain the difference in frost hardiness between roots and shoots. The relative water content is up to three times higher in roots than in aboveground organs. This higher water content would be associated with reduced hardiness, presumably because it increases the risk of deleterious ice formation (Burke et�al. 1976, Levitt 1980, Bigras et�al. 1989, Pearce 1999). The insulation provided by soil and the consequent higher fall and winter temperatures to which roots are exposed could reduce the maximum frost hardiness achievable. Indeed, Magness (1929) observed that *Malus* sp. roots exposed to the air could achieve the same degree of frost hardiness than the shoot. Contrary to this, Ryypp� et�al. (2008) reported that *P. sylvestris* roots were less frost hardy than aboveground organs, even when they were exposed to the same temperatures, and similar results are reported for horticultural woody species (Pellett 1971).

## Environmental Signals Inducing Cold Acclimation and Frost Hardening

### Cold acclimation and frost hardening

In nature, cold acclimation is initiated in late autumn or early winter. This period is characterized by a reduction in day length and lowering temperatures, which act as environmental signals inducing cold acclimation at the whole plant level. Cold acclimation can also be induced under laboratory conditions, simply by exposing plants to low temperatures (2–8�C) and/or reduced day length. Interestingly, it has been observed that for some species, exposure to cold temperature was enough to induce full acclimation, indicating that cold temperature is the primary cue for cold hardening for these species (Li et�al. 2005, Ryypp� et�al. 2008, Sepp�nen et�al. 2018).

Different environmental cues induce cold acclimation in aerial and hypogeal organs. In the aboveground organs, cold acclimation can be divided into two or three phases depending on the species. The first phase, which is usually induced by shortening of the light period, results in the reduction of growth and the accumulation of starch as a storage resource in the roots (Hashimoto and Komatsu 2007, Degand et�al. 2009, Dumont et�al. 2011). The second phase, which is promoted by temperatures close to 0�C, is characterized by the de novo production of membrane lipids, proteins and metabolites involved in frost hardiness (Livingston and Henson 1998, Beck et�al. 2004). The second hardening phase is usually faster than the first one, e.g. Beck *et al.* (2004) reported a hardening rate of 0.3�C per day during the first phase and 0.9�C per day during the second phase. A third acclimation stage specific to woody species that are able to resist extremely low temperatures can be triggered by exposure to temperatures between −30�C and −50�C. In these plants, water vitrifies inside the cell, which protects them from the fatal consequences of cytoplasm crystallization (Ruelland et�al. 2009, Strimbeck et�al. 2015).

The root zone temperature seems to be the main environmental signal inducing root hardening (Ryypp� et�al. 2008, Sakai and Larcher 1987). Contrary to most studies, no seasonal impact on root frost hardiness was observed in walnut tree (*Juglans regia*) using electrolyte leakage (Charrier et�al. 2013). Aside from the temperature, the photoperiod could also play an indirect role in root cold acclimation via a change in the carbohydrates source–sink relationship between above- and belowground organs (Smit-Spinks et�al. 1985). Two phases of cold acclimation have been observed in the roots of *Juniperus chinensis* and *P. sylvestris* (Bigras et�al. 1989, Ryypp� et�al. 2008). The first phase takes place at relatively high temperatures (between 5�C and 10�C) and results in reduced root growth and a slight increase in frost tolerance. During the second phase, which is promoted by temperatures close to 1�C, root growth totally stops and frost hardiness further increases.

Whether perennial roots enter true dormancy [endodormancy, as defined by Lang et�al. (1987)] or remain quiescent (ecodormant) once their growth has stopped has been debated. For some authors, root growth slows down during autumn and roots then enter true dormancy, i.e. their dormancy is controlled by internal factors and they do not immediately resume growth if they are exposed to favorable conditions alone (Romberger 1963, Johnson-Flanagan and Owens 1985). For others, roots become quiescent rather than truly dormant: as temperature drops below a certain threshold, roots stop to grow, but they resume growth as soon as favorable conditions arise (Green and Fuchigami 1985). Since the 1980s, no definitive answer has been given (Bigras and Dumais 2005). Although the knowledge on molecular mechanisms involved in quiescence and dormancy has increased with the advent of cost-effective transcriptomic techniques, we still do not know what are the precise molecular controls that differ between quiescent and dormant plant cells (Considine and Considine 2016). However, recently, Hong et�al. (2017) reported that chilling causes DNA damage and induces protective death of columella stem cell daughters. This protective death improved roots’ ability to overcome cold stress and to resume growth when optimal temperatures were restored.

### Deacclimation and dehardening

Temperature is the main environmental factor triggering dehardening of above- and belowground organs (Beck et�al. 2004, Ryypp� et�al. 2008). Some studies indicate that root dehardening is also affected by the photoperiod (Tinus et�al. 2000), although the generalization of this influence is debated (McKay 1994, Beck et�al. 2004). Dehardening might also depend on the duration of the quiescence period (�gren 1997, Kalberer et�al. 2006). �gren (1997) observed that during mild winters, Norwegian *P. sylvestris* partially dehardened due to the respiratory consumption of soluble carbohydrates reserves rather than because of the elevated temperature. In general, dehardening is faster than hardening and a significant degree of frost hardiness can be lost in a few hours to days when temperature increases (Taulavuori et�al. 2002, Cunningham et�al. 2003, Kalberer et�al. 2006). For example, while it takes 14 d to gain 5�C of cold hardiness for *Deschampsia antarctica*, this 5�C of frost hardiness is lost in 7 d (Chew et�al. 2012). Similarly, bilberries (*Vaccinum myrtillus*) exposed to 5�C in mid-winter lost >30�C of frost hardiness in 7 d (Taulavuori et�al. 2002). Deacclimation dynamics are linked to temperature, and plants deharden faster when they are exposed to higher temperatures (Taulavuori et�al. 2002, Charrier et�al. 2011). When plants completely deharden, their capacity to subsequently reharden diminishes or can even be lost (�gren 1997, Kalberer et�al. 2006).

As roots and shoots respond to different environmental stimuli, they do not harden at the same time, i.e. roots begin to cold acclimate later and deharden earlier than aerial parts (Bigras and Colombo 2001, McKay 1994). Roots need to stay biologically active for a longer time than shoots to ensure water provision. However, during late winter/early spring warm spells, the root zone temperature can remain lower than the air temperature, which results in limited water supply for aboveground organs and can lead to aboveground dehydration and reduced shoot productivity (Ryypp� et�al. 1998). The severity of dehydration stress depends on the delay between the beginning of the warm spell and soil thawing. In an experiment carried out in growth chambers, Repo *et al.* (2005) observed that exposure of *P. sylvestris* to a warm air temperature and frozen soil resulted in the death of the plants in just 2 weeks.

## Metabolic Responses

### Cold is perceived at the cellular level

‘Molecular perception of cold and signal transduction’ are required to integrate the environmental signals perceived by the roots and trigger the metabolic responses leading to cold acclimation. These metabolic responses include the accumulation of a variety of osmoprotectants, AFPs, antioxidants and stabilizing proteins such as chaperones, dehydrins (DHNs) and heat shock proteins (HSPs) (Fig.�3). Most studies on cold perception and signal transduction have been done on leaves and seedlings. From these, it appears that the changes in temperature are sensed by the membrane and cytoskeleton, which induce an influx of calcium that triggers downstream responses. Among the downstream processes, the signal transduction pathway includes C-repeat binding factors/dehydration-responsive element-binding factor (*CBFs/DREB*)-dependent and *CBFs*-independent pathways that lead to the induction of cold-responsive (*COR*) genes involved in cold acclimation.

**Fig. 3:**
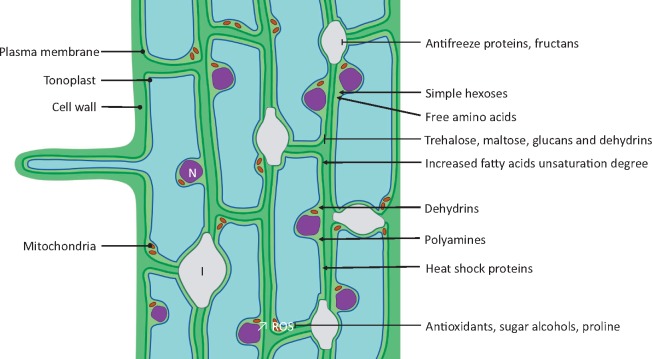
Protective mechanisms put in place by freezing-tolerant roots. The plasma membrane is the main site of damage during frost-induced stress. During intense osmotic stress, the plasma membrane and the tonoplast can come into close contact and their lipid bilayers can shift from a lamellar to a hexagonal II phase, leading to cellular leakage. The cell wall plays an important role in frost tolerance, and its strengthening can lead to increased frost tolerance. Antifreeze proteins and fructans decrease the expansion of ice crystal in the apoplast and inhibit the formation of icicles. Simple hexoses and free amino acids accumulate in the cytoplasm where they counterbalance the negative osmotic pressure induced by the presence of ice in the extracellular matrix. Dehydrins, heat shock proteins and polyamines stabilize the membranes and inhibit protein and mRNA denaturation. Trehalose, maltose and glucans stabilize the membranes and inhibit membrane fusion. Antioxidant concentrations increase and counter the increased ROS production with the help of proline and ROS-scavenging sugar alcohols, i.e. raffinose, galactinol, sorbitol. I, ice crystal; N, nucleus; ↗ ROS, increased ROS production.

More precisely, plants primarily perceive temperature fluctuations due to changes in membrane fluidity (Plieth et�al. 1999). Cold exposure leads to a decreased membrane fluidity, and cold acclimation can be artificially induced at 25�C with membrane-rigidifying agents, while membrane-fluidizing agents strongly inhibit cold transduction pathways, even when exposed to 4�C (Orvar et�al. 2000, Sangwan et�al. 2001, Furuya et�al. 2014).

During exposure to low temperatures, the plant cytoskeleton undergoes a rearrangement that is also linked to the induction of genes related to cold acclimation. Treatment of *Brassica napus* leaves and *A. thaliana* cell suspensions with microtubule and filament stabilizers inhibited the expression of *BN115* and *cas30*, while applying a microtubule and filament dispersant promoted the expression of the same genes (Orvar et�al. 2000, Sangwan et�al. 2001). Interestingly, Wang et�al. (2019) observed that while *Vitis rupestris* microtubules partially disassemble when they are exposed to chilling temperatures (8�C), they totally disassemble when they are cold-shocked (27–0�C). In addition, they showed that cells treated with taxol (a microtubule stabilizer) were able to withstand cold shock as well as cells that were cold-acclimated at 8�C for 72 h.

The increased membrane rigidity and cytoskeleton rearrangement trigger a fast and transient increase in cytosolic-free calcium concentration ([Ca^2+^]_c_) [cf. Knight and Knight (2012) for an extensive review]. Using aequorin, a photoprotein that indicates fluctuations in [Ca^2+^]_c_, Knight et al. (1991) observed that exposure of *Nicotiana plumbaginifolia* seedlings to 5�C and 0�C caused a strong transient increase in [Ca^2+^]_c_. No such increase was triggered when the seedlings were exposed to 10�C, 20�C or 40�C. Using similar experimental setup, Campbell et�al. (1996) observed that under gradual cooling (0.1�C s^−^^1^), the increase in [Ca^2+^]_c_ occurs at 8–10�C higher in roots of *N. plumbaginifolia* than in leaves of *N. plumbaginifolia*. This is expected as, under natural conditions, the roots are buffered against thermal variations by the soil; therefore, any change in temperature could be more significant to the roots than to the aboveground organs. Plieth et�al. (1999), who worked with intact root systems isolated from hydroponically grown *A. thaliana* expressing aequorin, observed that although the magnitude of the [Ca^2+^]_c_ elevation mainly depends on the cooling rate, the absolute temperature to which plants are exposed also has an impact. Higher cooling rate increases the magnitude of the cytosolic calcium influx, and lower absolute temperatures increase the ability of roots to respond to cold. While often used in the laboratories to study the response of plants to low temperatures, cold shocks at the root level are unrealistic under natural conditions as the roots are thermo-buffered by the soil. Plieth et�al. (1999) could not detect any [Ca^2+^]_c_ increase in roots of *A. thaliana* at cooling rate under 0.003�C s^−^^1^ (10.8�C h^−^^1^). However, it has been shown in *A. thaliana* cell suspensions that a calcium flux is required for cold acclimation and frost hardening (Monroy et�al. 1993). Therefore, it cannot be excluded that the calcium flux is localized closed to the cytosolic face of the plasma membrane or of the tonoplast (Knight and Knight 2012). The elevation of [Ca^2+^]_c_ has been shown to be a main determinant in the induction of *COR* genes (Knight et�al. 1996).

In addition to the increase in cytosolic-free calcium concentration, a rapid transient increase in phosphatidic acid has also been observed in plant exposed to cold and frost (Arisz et�al. 2013, Tan et�al. 2018). This increase in phosphatidic acid is generated through diacylglycerol kinase. In barley (*Hordeum vulgare*), the basal activity of diacylglycerol kinase is higher in the leaves than in the roots. However, short-term (3 h) cold exposure nearly doubles its activity in the roots but strongly reduces it in the leaves (Peppino Margutti et�al. 2018). The phosphatidic acid increase in barley roots has been linked to proline and ROS accumulation (Peppino Margutti et�al. 2018). Recently, it has been observed that acyl-coenzyme A:diacylglycerol acyltransferase, which catalyzes the conversion of diacylglycerol to triacylglycerol and thereby limits phosphatidic acid production and its possible deleterious effect on the membrane, is induced in the roots of *A. thaliana* exposed to frost (Tan et�al. 2018). The signal transduction pathway after phosphatidic acid production is not precisely known; however, it seems to imply a *CBF*-independent pathway (Tan et�al. 2018).

The calcium signal is then integrated by various calcium/calmodulin-binding proteins. In Arabidopsis roots, calcium activates a root plasma membrane calcium/calmodulin-regulated receptor-like kinase (CRLK1) (Yang et�al. 2010), which in turn activates the MEKK1–MKK2–MPK4 kinase cascade (Furuya et�al. 2014). Calcium also activates a calcium sensor-associated protein kinase (CIPK3) (Kim et�al. 2003) and other calcium-dependent protein kinases. Eventually, these protein kinase cascades lead to the phosphorylation of ICE1 and ICE2 (inducers of *CBF* expressions 1 and 2), which in turn activate the *CBFs* (Kim et�al. 2015).

*CBFs* are transcription factors with an AP2/ERF domain, which bind to the C-repeat element. This element consist of the five-base sequence CCGAC and is found in single or multiple repeats in the promoting region of many *COR* genes (Stockinger et�al. 1997, Jia et�al. 2016). In *A. thaliana*, three *CBFs* (*CBF1–3*) are found in tandem array on chromosome 4. *CBF1* overexpression has been shown to induce *COR* genes and to increase frost hardiness (Jaglo-Ottosen et�al. 1998), while ectopic expression of an apple *CBF* in peach resulted in increased hardiness and short-day induced dormancy (Wisniewski et�al. 2011). Detailed functional characterization among others indicates that *CBF1–3* are at least partially redundant (Gilmour et�al. 2004, Jia et�al. 2016). *CBF2* could have a fine-tuning role in cold acclimation as while it represses *CBF1* and *CBF3* expressions (Novillo et�al. 2004), an *A. thaliana* ecotypes having a nonfunctional *CBF2* have reduced cold acclimation capacity compared with ecotypes having a functional *CBF2* (Park et�al. 2018). Interestingly, although *CBFs* regulate only approximately 10% of the *COR* genes, triple *cbfs* mutant lost most of their frost-hardening capacity, suggesting a pivotal role of the *CBF*-dependent pathway in frost hardening (Jia et�al. 2016, Park et�al. 2018).

Once produced, the stability of CBFs proteins is modulated by various effectors. For example, the cold-induced plasma membrane protein CRPK1 (cold-responsive protein kinase 1) can phosphorylate a 14-3-3 protein, which downregulates CBFs’ activity (Liu et�al. 2017). Conversely, the cold-induced OST1-phosphorylated BTF3L (basic transcription factor 3-like) protein upregulates CBFs’ activity (Ding et�al. 2018).

There is a crosstalk between the signal transduction pathways of different stresses. For example, both the drought and oxidative stress signal transduction pathway imply the MEKK1–MKK2–MPK4 protein kinase cascade. Similarly, abscisic acid (ABA), a hormone traditionally associated with drought stress, can also activate CIPK3 (Kim et�al. 2003) and MYB96, a transcription factor upstream of *CBFs* (Lee and Seo 2015).

### Membranes are the primary sites of frost injury

One of the first effects of frost injury is an increased leakage of electrolytes from damaged cells to the apoplast (Ryypp� et�al. 2008). This makes the measurement of electrolyte leakage one of the primary tools for estimating cold temperature damage and a marker for frost hardening and dehardening (Pagter et�al. 2014). The sources of damage to the membranes are multiple. To begin, ROS can degrade membrane lipids through peroxidation. During lipid peroxidation, an ROS reacts with a hydrogen atom from a fatty acid tail, forming a fatty acid radical. This fatty acid radical can react with other fatty acid tails (therefore propagating the reaction), with itself (forming a cyclic peroxide) or with antioxidants (such as α-tocopherol, glutathione and ascorbic acid, thereby terminating the reaction) (Nayyar and Chander 2004). Lipid peroxidation mostly affects polyunsaturated fatty acids that possess more reactive hydrogen atoms.

Another effect of lowering temperatures is a shift in the phase of the lipid bilayer, from a liquid-ordered phase to a solid-gel state. The phase transition temperature is function of the number of carbon atoms present in the tail and the number of double bonds (higher unsaturation degree lowers the phase transition temperature). Phase transition reduces water diffusion through the membrane (Eze 1991) and changes lipid–protein interactions (Iivonen et�al. 2004). To tackle the reduction in water diffusion, the induction of roots aquaporins (proteins regulating water flux across the membrane) has been observed in roots of *Zea mays* exposed to cold temperatures (Aroca et�al. 2005). An increase in the fatty acid unsaturation has been observed in the roots of different cold-exposed plant species including *Medicago sativa* (Gerloff et�al. 1966), *H. vulgare* (Peppino Margutti et�al. 2019), *B. napus* (Smolenska and Kuiper 1977), *P. sylvestris* (Iivonen et�al. 2004) and *Glycine max* (Markhart et�al. 1980). It is important to emphasize that this change in the degree of unsaturation of fatty acids is largely due to the formation of new roots rather than the dehydrogenation of fatty acids contained in older roots (Markhart et�al. 1980).

In addition, frost changes the configuration of lipid bilayer. As frost-induced dehydration stress increases, membranes are brought in closer proximity and tend to shift from a lamellar to a hexagonal II phase. Hexagonal II phase membranes are characterized by the formation of inverted cylindrical micelles between the two outermost membranes. This transition is favored by a higher unsaturation degree of fatty acids, a decreased water content, a higher amount of intracellular membranes, and the presence of phosphatidylethanolamine and sterols in the bilipid membrane (Uemura et�al. 1995). Smolenska and Kuiper (1977) observed a reduction in the proportion of phosphatidylcholine (30%) and phosphatidylethanolamine (20%) during the cold acclimation of *B. napus* roots, while a 60% increase in an unidentified phospholipid was recorded. Interestingly, while roots of barley contain more phospholipids than leaves, long exposure to cold decreases the phospholipid and glycerolipid content in the roots but not in the leaves (Peppino Margutti et�al. 2019). A decrease in free sterols has been observed in cold-acclimated oat leaves, while no changes were observed in rye leaves (Laque et�al. 2016) and a decrease in the sitosterol (a sterol restricting the motion of fatty acid tails) level has been observed in *P. sylvestris* roots during deacclimation (Iivonen et�al. 2004). This transition from a lamellar to the hexagonal II phase may be more frequent in roots as they have a higher sterol content (Rochester et�al. 1987), but on the other hand, they also possess less intracellular membranes.

The intense dehydration also provokes expansion-induced lysis. Negative osmotic pressure induces a net movement of water towards the extracellular space and thus reduces cell volume. In the protoplast of nonacclimated cells, this reduction in cell volume provokes the invagination of the plasma membrane and the formation of endocytic vesicles, resulting in a loss of surface of the plasma membrane. Upon rewarming, melted water from the extracellular space goes back to the cell, which causes the burst of the cell before it can regain its former volume. In protoplasts of cold-acclimated cells, the reduction in volume induces the formation of exocytotic extrusions, which does not reduce membrane surface and does not provoke cellular burst upon rewarming (Uemura et�al. 1995, Ruelland et�al. 2009). The precise mechanism favoring the formation of exocytotic extrusions rather than endocytic vesicles is unknown. It has been observed that the incorporation of mono- and di-unsaturated fatty acids during cold acclimation promoted exocytotic extrusion (Uemura et�al. 1995).

Finally, membranes are also exposed to the destructive effects of extracellular ice crystal formation. The formation of bigger (and more harmful) ice crystals during ice recrystallization is favored by repeated freeze–thaw cycles (Antikainen et�al. 1996, Zachariassen and Kristiansen 2000) and is tackled by several mechanisms mentioned below.

### Cell walls mediate interaction with the environment

Several reports in literature have described an active role of the cell walls in response to abiotic constraints [reviewed by Le Gall et�al. (2015)]. The space between adjacent cell walls is thought to be the main site of ice nucleation as it is where most of the INAs are found (Goldstein and Nobel 1994). Cell wall plasticity is linked with frost resistance, and in several species, cold acclimation has been reported to induce cell wall strengthening (Arias et�al. 2015). This strengthening could reduce ice propagation (Sasidharan et�al. 2011) and damage to the cell membranes during thawing (Zhang et�al. 2016). Some plants also developed strategies to cope with ice propagation, under the form of faults (flexible junctions that can expand and accommodate the formed ice crystals) connecting surface tissues with internal ones and anchorages overlying the vascular bundles (McCully et�al. 2004). Furthermore, the anatomy of the conductive system plays a pivotal role in frost tolerance: in a study analyzing Patagonian tree shrubs, species with smaller xylem vessels showed enhanced stem supercooling ability, with ice nucleating at lower temperatures (Zhang et�al. 2016). Interestingly, species adapted to lower temperatures had larger xylem vessels and therefore tended to freeze at higher sub-zero temperatures.

Low temperatures also affect cell wall composition. For example, it has been observed that the roots of cold-sensitive chicory (*Cichorium intybus*) varieties exposed to low temperature had lower pectin methylesterase (PME) activity than cold-tolerant varieties (Thonar et�al. 2006). Similarly, overexpression of a PME inhibitor was found to reduce the freezing tolerance of *A. thaliana* roots (Chen et�al. 2018).

Different responses to low temperatures have been reported in gene expression studies: e.g. low temperature stress represses genes involved in secondary cell wall biosynthesis and lignification in the roots of alfalfa (*Medicago sativa*) (Behr et�al. 2015). In stems of alfalfa exposed to cold, the genes encoding secondary cell wall cellulose synthases are downregulated (Guerriero et�al. 2014). Leaves of frost-tolerant *Miscanthus* clone showed an increased activity of hydroxycinnamyl alcohol dehydrogenase and phenylalanine ammonia lyase, thereby suggesting shunting carbon towards the synthesis of phenylpropanoids, which, however, did not result in an increased lignin content (Domon et�al. 2013). It is likely that species with different cell wall composition rely on distinct cell wall modifications to counter low temperature stress. The use of multi-pronged approaches coupling -*omics* with immunohistochemical and chemical characterization of the cell wall will contribute to shed light on the complex mechanisms involved

### Frost disturbs ROS homeostasis

‘The antioxidant system’ has to adapt to the unbalance of the redox homeostasis caused by frost stress. ROS such as superoxide anion, hydrogen peroxide and hydroxyl radical are generated by plants as part of their metabolism, but exposure to biotic and abiotic stresses leads to an increased accumulation of ROS (Foyer and Noctor 2009, Ruelland et�al. 2009). ROS have a dual function in plants, i.e. they act as secondary messengers in signal transduction at low concentrations (Foyer and Noctor 2009, Berni et�al. 2018), while at higher concentrations, they cause oxidative damage ultimately leading to cell death (Rhoads et�al. 2006). In roots and other heterotrophic tissues, ROS are mainly produced by the mitochondrial electron transport chain complexes I and III. Complex III releases ROS in the mitochondrial intermembrane space from where they can migrate to the cytosol (Rhoads et�al. 2006). Peroxisomes are another ROS source through lipid beta-oxidation, although their precise impact on the total ROS production in heterotrophic plant cells is unknown (Miller et�al. 2010).

Under nonstressing conditions, there is a balance between ROS production and ROS scavenging. However, cold disrupts this balance by inducing the rigidification of mitochondrial membranes (Saha et�al. 2015) and provoking the loss of complex IV (Prasad et�al. 1994). These events impede electron transfer reactions, leading to increased ROS production. Since cold directly impacts enzymatic activities, including that of proteins contributing to the maintenance of the cellular redox balance, this is exacerbated by freezing temperatures (Baek and Skinner 2012).

To alleviate the oxidative challenge induced by low temperatures, plants can either reduce ROS production or increase ROS-scavenging capacities. The level of ROS produced in the roots during cold stress can be reduced by the induction of the mitochondrial alternative oxidase pathway, alternative NAD(P)H dehydrogenases or uncoupling proteins (Heidarvand et�al. 2017). Cold-acclimated plants and cold-resistant cultivars have a higher level of alternative oxidase than nonacclimated plants and cold-sensitive cultivars (Heidarvand et�al. 2017). Several enzymatic and nonenzymatic antioxidants are cold-induced, and their concentrations have been linked to cold hardiness. Table�1 summarizes some of the changes in the activities and concentration of different antioxidants observed in the roots of cold-acclimating plants. In addition, cysteine synthase, an enzyme resulting in the production of cysteine, an amino acid essential in glutathione synthesis, has been found to be induced in cold-exposed rice roots (Lee et�al. 2009). In an ‘omics’ study comparing roots and leaves of cold-acclimating strawberries (*Fragaria* � *ananassa* cv. Korona), Koehler et�al. (2015) observed an initial decrease in cysteine in both the roots and the leaves, followed by a subsequent accumulation of cysteine in the roots but not in the leaves.

**Table 1:** Changes in the activities and concentration of various enzymes and metabolites involved in the maintenance of the redox homeostasis in the roots of cold-acclimated plants

	*Cicer arietinum* (Nayyar and Chander 2004)	*Oriza sativa* (Kuk et�al. 2003)	*M. sativa* (Gerloff et�al. 1967)	*Medicago ciliaris* (Yahia et�al. 2016)	*Zea mays* (Prasad et�al. 1994)	*Cichorium intybus* (Degand et�al. 2009)	*Pinus banksiana* (Zhao and Blumwald 1998)	*Panax ginseng* (Devi et�al. 2012)	*Triticum aestivum* (Scebba et�al. 1999)	*Glycine max* (Posmyk et�al. 2005)	*Lens culinaris* (�ktem et�al. 2008)
GSH	+						+++				
AsA	++										
APX	+	+				+	++	++			++
GuPX			+	+	++		+	+	+++		
GSR		+					+				
SOD	+	+				+			+++	0	−
CAT	++	++	+		+	+		++	+++	+	0

GSH, glutathione; AsA, ascorbic acid; APX, ascorbate peroxidase; GuPX, guaiacol peroxidase; GlPX, glutathione peroxidase; GSR, glutathione reductase; SOD, superoxide dismutase; CAT, catalase. +++ (dark green), >4-fold increase; ++ (green), between 2- and 4-fold increase; + (light green), <2-fold increase; 0 (yellow), no significant change; − (orange), <2-fold change decrease.

### Carbohydrates have multiple protective properties

‘Carbohydrates and sugar alcohols’ are associated with cold acclimation and frost hardiness and have protective effects. (i) They are compatible solutes, i.e. low molecular weight soluble molecules that accumulate at high concentration with no cytotoxic effects. As such, they relieve osmotic stress caused by the frost-induced lower water potential of extracellular fluids. (ii) They lower the freezing point in a colligative way and appear to be determinant in the water vitrification process (Wolfe and Bryant 1999, Strimbeck et�al. 2015). (iii) Sugars (defined here as oligosaccharides) and sugar alcohols stabilize membranes and proteins during intense dehydration. While it was thought that sugar molecules replaced water molecules in the hydration shell of proteins (Xin and Browse 2000), Di Gioacchino et�al. (2018) observed with neutron diffraction that trehalose forms a protective shell that traps water molecules at the surface of proteins, thereby avoiding dehydration-induced denaturation. Sugars seem to protect lipid bilayers from dehydration with a similar mechanism (Konov et�al. 2015). By providing a protective shell, sugar molecules limit membrane fusion and protein aggregation by keeping intracellular complexes apart from each other (Mensink et�al. 2017). Trehalose and maltose are particularly effective in stabilizing membranes and proteins and inhibit membrane fusion, even at low concentrations (Wolfe and Bryant 1999). Glucans also inhibit membrane fusion, with increased capacity as the polymerization degree increases. In addition to these protective properties, sugars lower the gel-to-liquid crystalline phase transition temperature of lipid bilayers, hence allowing them to stay functional at lower temperatures. A decrease in the phase transition temperature of >30�C in liposomes in the presence of sucrose and low molecular weight raffinose family oligosaccharides (RFOs) and >20�C in the presence of fructans and glucans was reported (Hincha et�al. 2002, Hincha et�al. 2003). (iv) The accumulation of simple carbohydrates has a predominant role in tolerance to extremely low temperatures (under −40�C) in woody plants by favoring water vitrification rather than crystallization (Mensink et�al. 2017). Trehalose has a higher glass transition temperature and is less prone to crystallization than most carbohydrates, making it particularly important for tolerance to extremely low temperatures (Strimbeck et�al. 2015, Mensink et�al. 2017). Sucrose tends to crystallize at high concentrations, but small amounts of RFOs reduce sucrose’s propensity to crystalize (Wolfe and Bryant 1999). In addition, fructans interact with ice crystals and change their growth pattern and morphology (Shearman et�al. 1973). (v) Sugars and sugar alcohols protect the cells from oxidative damage through active ROS scavenging (Keunen et�al. 2013). Among them, raffinose, galactinol, sorbitol, mannitol and myo-inositol appear to be the most effective free radical scavengers (Morsy et�al. 2007, Folgado et�al. 2015).

Sugar accumulation is highly correlated with frost hardiness. Hardier plants accumulate sugars earlier and to a higher extent than cold-susceptible plants (Cunningham and Volenec 1998, Sepp�nen et�al. 2018). During cold acclimation, sugar accumulation takes place in two phases. First, shortening of the photoperiod triggers a change in the allocation of photosynthates from shoot growth to nonstructural carbohydrate reserves in the large roots, where it accumulates mainly under the form of starch (Cunningham and Volenec 1998, Sepp�nen et�al. 2018). This increase in starch concentration is poorly and/or negatively correlated with frost hardiness (Tinus et�al. 2000, Kalberer et�al. 2006, Sepp�nen et�al. 2018). During the second phase, this starch is hydrolyzed into simple sugars, resulting in an increased frost hardiness (Lennartsson and �gren 2002, Koehler et�al. 2015, Sepp�nen et�al. 2018). The environmental signal triggering starch hydrolysis is not yet deciphered: for long it was thought that lowering temperatures triggered this response, but enzymes involved in starch degradation are already induced before temperatures dropped in some poplar species (Guy et�al. 2008).

In alfalfa roots, the starch content increases with shortening days and is at its maximum by the end of September when the level of soluble sugar is at its minimum (Cunningham and Volenec 1998, Sepp�nen et�al. 2018). With lowering temperature, sucrose and RFOs accumulate while starch, glucose and fructose concentrations decrease. Interestingly, cold hardiness is more correlated with the accumulation of RFOs (*r*� = 0.92) than with glucose and sucrose (*r*� = 0.48) in the roots of alfalfa (Cunningham et�al. 2003). Similarly, in poplar roots, frost hardiness is better correlated with trehalose and RFOs content than with glucose, fructose and sucrose concentrations (Regier et�al. 2010). Furthermore, *Picea abies* and *Carthamus tinctorius* roots proportionally accumulate more raffinose than sucrose when exposed to chilling temperatures (Wiemken and Ineichen 1993, Landry et�al. 2017). Koehler et�al. (2015) observed the accumulation of raffinose, galactinol and glucose in both the roots and the leaves of cold-acclimating strawberry, while fructose accumulated only in the roots, and glucose only in the leaves.

Seven genes implicated in trehalose synthesis *AtTPS2*, *AtTPS3*, *AtTPPD* and *AtTPPF* (Iordachescu and Imai 2008), and *AtTPPA*, *AtTPPG* and *AtTPS11* (Kreps et�al. 2002) were reported to be up to 48-fold more expressed in cold-exposed *A. thaliana* roots. Kreps et�al. (2002) also detected a 65-fold increase in the *AtBAM3* transcript, coding for a β-amylase involved in maltose accumulation, >20-fold increase in two galactinol synthase transcripts (involved in RFOs synthesis) and an up to 7-fold increase in β-glucosidase mRNA levels in the roots of cold-exposed *A. thaliana*. Galactinol synthase transcripts were found to increase in cold-acclimating alfalfa roots, and this increase in transcript level was followed 1 week later by the increased accumulation of RFOs (Cunningham et�al. 2003). Degand et�al. (2009) observed an important increase in fructan:fructan 1-fructosyl level, involved in fructan synthesis, in cold-acclimated chicory roots.

### Polyamines alleviate freezing stress

Polyamines (PAs) are small ubiquitous, polycationic aliphatic molecules, having at least two amino groups. PAs are positively charged at physiological pH and have been shown to bind to negatively charged proteins, acidic phospholipids and nucleic acids, stabilizing them. In nonstressed plants, PAs have a wide array of functions including the regulation of cell proliferation, differentiation, morphogenesis and senescence (Minocha et�al. 2014). Increased levels of PAs have been observed in plants exposed to various stresses, including chilling, and are usually associated with increased stress tolerance (Saha et�al. 2015, Strimbeck et�al. 2015). In addition to their stabilizing properties, PAs have radical-scavenging properties (Gupta and Huang 2014). However, this is debated as Langerbartels et�al. (1991) observed that while conjugated PAs have high radical-scavenging activities, radical-scavenging properties of free PAs are rather poor. Furthermore, anabolism and oxidative catabolism of PAs were reported to substantially increase H_2_O_2_ levels (Tang and Newton 2005, Minocha et�al. 2014).

In plants, the most commonly found PAs are putrescine (Put; a diamine), spermidine (Spd; a triamine) and spermine (Spm; a tetraamine) (Minocha et�al. 2014). Put is synthesized through decarboxylation of either arginine or ornithine and is used as a substrate to generate higher PAs. Spd and Spm are synthesized through the addition of an aminopropyl residue on Put and Spd, catalyzed by Spd and Spm synthase, respectively (Lutts et�al. 2013). Other PAs have been found in stressed plants such as cadaverine (in Leguminosae, Solanaceae and Graminae) (Lutts et�al. 2013), cadiamine (in Leguminosae) (Smith 1975), agmatine (R�cz et�al. 1996), thermospermine (a spermine isomer) (Saha et�al. 2015) and various conjugated PAs (Guo et�al. 2014).

Roots and shoots have different PAs’ accumulation patterns. Koehler et�al. (2015) observed that ornithine accumulates in the roots of strawberries during cold acclimation but not in their leaves, while putrescine accumulates in the leaves but not in the roots. In addition, methionine (a precursor of spermidine and spermine) accumulated in the leaves but not in the roots. Lee et�al. (1997) reported an increase in Put, Spd and Spm in cold-exposed *Oryza sativa* shoots while only Put accumulated in the roots. Analogous patterns are observed in the roots of four wheat (*T. aestivum*) varieties (R�cz et�al. 1996). In addition to the increase in Put content, cold-exposed wheat roots accumulate agmatine (R�cz et�al. 1996).

In a transcriptomic study, Yang et�al. (2015) reported that genes involved in Put biosynthesis were cold-induced in both the shoots and roots of chilling-tolerant rice varieties, while Spd- and Spm-related genes were only induced in the shoot. Guo et�al. (2014) observed no *MfSAMS1* transcript in roots of cold-exposed *M. sativa*. *MfSAMS1* codes for *S*-adenosylmethionine synthetase, which catalyzes, among others, the formation of *S*-adenosylmethionine, the precursor of the aminopropyl residue added on Put and Spd to give Spd and Spm, respectively. Similar changes were reported in the roots and leaves of cold-acclimating strawberries (Koehler et�al. 2015). Conversely Imai et�al. (2004) observed an 8-fold increase in the level of *OsSPDS2* mRNA (coding a putative 42-kDa spermidine synthase) in *O. sativa* roots exposed 4 d to low temperatures and Kreps et�al. (2002) reported a 2.1-fold increase in the transcript level of a spermine synthase in cold-exposed *A. thaliana* roots. Tang and Newton (2005) observed that while exogenous application of Put increased the growth speed of *Pinus virginiana* root tips, Spd and Spm inhibited it.

Some free amino acids are also associated with cold exposure and frost hardiness. They act as compatible solutes and stabilize membranes and proteins in a way similar to sugars. One of the amino acids that has frequently been found to accumulate is proline (�ktem et�al. 2008, Yin et�al. 2017). In addition to its compatible solute properties, proline stabilizes polyribosomes and acts as an ROS scavenger and a pH and redox buffer (Hayat et�al. 2012). It also limits chilling injury at the whole plant level when exogenously applied to *Vigna radiata* seedlings (Posmyk and Janas 2007). Interestingly, *Picea asperata* roots, which accumulate high level of proline (typically the pioneer roots), are more resistant to freeze–thaw cycles than roots with increased ROS-scavenging capacity (the fibrous roots) (Yin et�al. 2017). In addition to proline, alanine and arginine have been found to accumulate in alfalfa roots (Wilding et�al. 1960) and glutamine and glycine betaine accumulation was observed in winter hardy *Beta vulgaris* roots (Loel and Hoffmann 2015). While the leaves of strawberries accumulate proline during cold acclimation, only a transient increase can be observed in their roots (Koehler et�al. 2015).

### AFPs change water freezing pattern

AFPs are proteins present in different cold-living organisms (plants, insects and arctic fish) that can inhibit and/or curb ice formation (Wisniewski and Fuller 1999; Griffith and Yaish 2004). AFPs work in a noncolligative way by binding to the lateral face of ice crystals during ice formation and growth. Typically, crystals formed in a solution containing AFPs are small and form hexagonal columns or hexagonal bipyramids, while in pure water, the ice crystals are wider and have a flat disk shape (Meyer et�al. 1999, Griffith and Yaish 2004).

Although fish, insect and plant AFPs have the same name and same mode of action, they differ in structure and protection strategies. Animal AFPs are typically flat and hydrophobic and create the thermal hysteresis (difference in temperature between the freezing point and the melting point) of 2–6�C (Griffith and Antikainen 1996). On the other hand, plant AFPs are characterized by multiple highly hydrophilic ice-binding domains in their amino acid sequences containing highly conserved asparagine residues spaced at a regular interval that is complementary to the prism plane of ice (Griffith and Yaish 2004). Their thermal hysteresis is low (from 0.2�C to 0.6�C), but they inhibit ice recrystallization occurring during repeated freeze–thaw cycles or when plants are exposed to low temperatures for an extended period of time (Meyer et�al. 1999, Wisniewski and Fuller 1999, Griffith and Yaish 2004).

Some plants AFPs’ sequences are homologous to the following three types of plant pathogenesis-related proteins: endochitinases, endo-β-1,3-glucanases and thaumatins (CLPs, GLPs and TLPs, respectively) (Antikainen et�al. 1996, Pearce 2001). This could indicate a double role in cold survival: reduce damage linked to frost and provide defense against psychrophilic pathogens (Griffith and Yaish 2004). Interestingly, Stressmann et�al. (2004) found that the chitinase and antifreeze activities of an AFP present in the apoplast of winter rye are tuned depending on the Ca^2+^ concentration. The antifreeze activity was lost when exposed to CaCl_2_ and recovered when adding a chelator. Conversely, the chitinase activity increased by up to 5-fold in the presence of Ca^2+^ ions.

AFPs are found in the roots of various cold-acclimated mono- and dicotyledonous angiosperms. Antikainen et�al. (1996) observed AFP-specific distribution pattern in *Secale cereale* roots. Three AFPs (a 30-kDa apoplastic CLP, a 72-kDa intracellular CLP and a 27-kDa intracellular TLP) accumulated in the epidermis of cold-acclimated roots, while only CLPs were found in the endodermis and vascular tissues of nonacclimated roots. An AFP has also been observed in the cold-acclimated taproot of *Daucus carota* (Meyer et�al. 1999, Smallwood et�al. 1999). This 332 amino acids long protein is, unlike most other AFPs, more closely related to polygalacturonase inhibitor protein than to CLP, GLP and TLP. It is a leucine-rich protein with a glycosylated N-terminal domain and is found in the apoplast. It inhibits ice recrystallization at concentrations as low as 150 �g ml^−1^, and its thermal hysteresis is of only 0.34�C (Meyer et�al. 1999). More recently, Chew et�al. (2012) found 60-, 700- and 1,000-fold increases in the transcript level of *DaIRIP8*, *DaIRIP1* and *DaIRIP4* (AFPs) in cold-acclimated *D. antarctica* roots.

### Dehydrins stabilize membranes and proteins

DHNs are a group of diverse proteins (ranging for 9–200 kDa) belonging to the late embryogenesis abundant protein group. DHNs are characterized by a repeated C-terminal lysine-rich consensus sequence known as the K-segment (Close 1997, Kosov� et�al. 2007, Hanin et�al. 2011). Many DHNs also have other conserved segments: the Y-segment (rich in tyrosine) found near the N-terminus and/or the S-segment (rich in serine), which can be phosphorylated as a signal for nuclear targeting (Hanin et�al. 2011). Some DHNs also possess a less conserved region rich in hydrophilic residues and glycine called the φ-segment (Close 1997). Close (1997) classified DHNs into the following five types depending on the segments they possess: K_*n*_, K_*n*_S, SK_*n*_, Y_2_K_*n*_ and Y_*n*_SK_2_. DHNs of the SK_*n*_ type are often induced by low temperature (Mingeot et�al. 2009). While some DHNs are ubiquitous, others seem to be organ and/or organelle specific (Hanin et�al. 2011). The abundance of DHNs is influenced by the hardening state of the plants, as for instance indicated by the decreased abundance of DHNs during the sigmoid dehardening process of *Hydrangea paniculata* bark and xylem (Pagter et�al. 2014).

In aqueous solution, DHNs adopt a random coil structure due to intermolecular hydrogen bonds with surrounding water molecules and a few intramolecular hydrogen bonds and are therefore classified as intrinsically disorder proteins (Kosov� et�al. 2007, Hanin et�al. 2011). The K-segment is predicted to form an amphipathic α-helical structure with negative charges at one side, hydrophobic residues at the opposite side and positive charges in between (Hanin et�al. 2011, Hara et�al. 2017). This K-segment is essential for protective activity as it interacts with the dehydrated surface of other proteins or with membranes and stabilizes them. They also prevent molecular aggregation and protein denaturation and inactivation (Hara et�al. 2017). In addition to this stabilizing property, it has been proposed that DHNs act as ‘space-fillers’ and keep cellular complexes at nonharmful distances (Strimbeck et�al. 2015). Furthermore, some DHNs have complementary roles to their stabilizing properties such as ROS scavenging (Hara et�al. 2004), metal binding and transport (Hara et�al. 2005) and antifreeze activity (Wisniewski et�al. 1999).

Nylander et�al. (2001) observed four (COR47, ERD14, ERD10/LTI29 and LTI30) DHNs in *A. thaliana* roots. With immunohistochemical localization, they showed that ERD10/LTI29 and ERD14 are expressed at a low level and observed the proteins in root tips and root vascular tissues of control plants. Upon low temperature exposure, the expression of *ERD10/LTI29*, *LTI30* and *ERD14* was induced and the corresponding proteins were found to accumulate in all root cells although to a higher extent in vascular tissues. This distribution pattern is similar to that of WCOR410, a membrane-binding DHN found in wheat (Danyluk et�al. 1998). The precise localization of COR47 could not be determined, but its transcript level increased in the roots after 3 h cold exposure. COR47 and ERD10/LTI29 were found to be cold specific as they are strongly induced by cold but only marginally by ABA or saline stress. An ERD14 homolog was also observed in cold-acclimated chicory roots along with CAS15, CiDHN1 and CiDHN2 and a 25-kDa DHN (Degand et�al. 2009, Mingeot et�al. 2009). DHNs also accumulate during winter quiescence and cold acclimation in the roots of woody plants (Kosov� et�al. 2007).

Other stabilizing proteins such as cold-induced HSPs [sometimes called cold shock proteins (CSPs)] and chaperones play an important role in cold resistance. HSPs are proteins constitutively expressed in all living organisms that are induced under stressful conditions. They were first discovered associated with the response to heat shock (hence their name) but have ever since been associated with other stress responses. The main function of HSPs is the role of molecular chaperones, helping for the folding of proteins and stabilizing their tertiary structure, thereby preventing molecular aggregation. However, some HSPs have also been linked to signaling protein degradation and targeting and others have been shown to bind to the 5′-untranslated region of some mRNAs, stabilizing them and enhancing their translation (Jacob et�al. 2017). Several HSPs are induced in roots exposed to cold. A CSP with RNA chaperone activity (AtCSP2) found in *A. thaliana* cold-exposed roots (Sasaki et�al. 2007) and different HSP70 (a family of HSP of 70 kDa), essential for protein folding, assembly and degradation (Jacob et�al. 2017), are cold-induced in roots of spinach (Anderson et�al. 1994), chicory (Degand et�al. 2009), rice (Lee et�al. 2009) and pea (Dumont et�al. 2011).

## Perspectives

Despite an increasing average global temperature, frost damage at the root level is likely to increase in the near future. This is mainly due to an increased weather variability and a reduction in the snow cover in winter, exposing roots to lower temperatures and to more freeze–thaw cycles. This is predicted to result in decreased agriculture and agroforestry productivity in temperate and colder climates (Kreyling et�al. 2012).

Several methods can be used to mitigate the deleterious effects of freezing temperatures on the roots and consequently on plant primary production. Direct compensation for the loss of snow cover with foam or an artificial cover to insulate the soil is currently done in Northern tree nurseries (Snyder and de Melo-Abreu 2005). Priming via chemicals or other stressors is another possibility to increase root frost hardiness (Posmyk and Janas 2007, Hossain et�al. 2018). Similarly, incubation of the seeds with growth-promoting bacteria has been shown to induce chilling and/or frost tolerance in the roots of young seedlings (Subramanian et�al. 2016). However, these two methods can only be implemented at small or medium scale and would need to be repeated every year to keep their protective effects, making them expensive on the long term.

Another solution is the breeding and/or selection of new varieties with improved frost tolerance at the root level. This can be done through the following two approaches: breeding and genetic engineering. The advantages are that it would be less costly on the long term and can be generally applied. Nevertheless, breeding is time consuming and while plants that are genetically engineered to constitutively overexpress *CBFs* are more frost resistant, they suffer from dwarfism and phenological changes (Sanghera et�al. 2011, Artlip et�al. 2016). These developmental defects are due to the *CBF*-induced accumulation of DELLA proteins, a family of nuclear growth repressors (Achard et�al. 2008). These unwanted pleiotropic effects can potentially be alleviated by simultaneously overexpressing growth-promoting genes or by engineering *DELLA* genes so that they are not activated by CBFs. Although such gene stacking has already been done to increase drought tolerance while reducing growth penalties, double transformants still have a primary production closer to *DREB1A*/*CBF3* overexpressors than to control plants (Kudo et�al. 2017). Similarly, the use of stress-inducible promotor to control the overexpression of *CBFs* can also lead to pleiotropic phenotypes in nonstressed plants, although the growth setback is less pronounced than in plants constitutively overexpressing *CBFs* (Morran et�al. 2011). Apart from *CBFs*, overexpression of *COR* genes has been done. The overexpression of AFPs in the roots of spring wheat resulted in increased root frost tolerance (Khanna and Daggard 2006). However, in a review on the topic of using AFPs to increase frost tolerance at the root or whole plant level, the authors concluded that none of these studies have resulted in a significant level of protection (Duman and Wisniewski 2014). To overcome these issues, the use of molecular markers could hasten breeding. Similarly, editing endogenous *CBFs* genes and/or downstream genes into variant sequences coming from more frost-tolerant species/varieties could remove the negative impacts of *CBFs*/*COR* overexpression on plants’ growth and productivity. However, few frost tolerance markers have been validated for the roots and, while it is known that cold differently impacts the molecular responses of roots and aboveground organs (Kreps et�al. 2002, Koehler et�al. 2015), the precise impact of cold on the root molecular response is poorly studied.

Future studies will also need to study the interaction existing between stresses. Indeed, under natural conditions, roots are likely exposed to more than one stress at the time (Mittler 2006). While some stresses, such as heat shock and drought, could potentially strengthen cold acclimation (Hossain et�al. 2018), others have unknown impact on root cold acclimation.

While there is extensive knowledge on the impact of freezing stress on aboveground organs, the mechanisms of root frost tolerance are largely unknown. This makes that progress in the generation of frost-tolerant plant crops, at the whole plant or root level, is limited. To attain this aim, a concerted effort of fundamental and agricultural research focusing on root frost tolerance is needed.

## Funding

This work was supported by the Luxembourg National Research Fund (FNR) [project X-press AFR PhD/17/SR 11634190].

## Disclosures

The authors have no conflicts of interest to declare.
